# Is befriending a possible intervention in people living with schizophrenia?

**DOI:** 10.3389/fpsyt.2025.1598355

**Published:** 2025-06-03

**Authors:** Felicia Iftene, Adriana Farcas, Simon O’Brien

**Affiliations:** Providence Care Hospital, Queen’s University, Kingston, ON, Canada

**Keywords:** schizophrenia, befriending, psychosocial measurements, schizoaffective disorder, intervention

## Abstract

**Introduction:**

Befriending is a non-specific intervention that may be valuable, cost-effective and easy to implement, complementing the complex therapeutic approach that schizophrenia requires.

**Objectives:**

This is a prospective, repeated-measures study design aimed to evaluate the possible clinical and functional changes in people living with schizophrenia undergoing 4 individual-basis, weekly Befriending sessions. The chart reviews supplemented the demographic information.

**Methods:**

Participants: 32 individuals with a diagnosis of schizophrenia or schizoaffective disorder were enrolled in this study. Specific psycho-social instruments were used to assess the possible clinical and/or functional changes post-intervention.

**Results/discussions:**

No statistically significant clinical improvement was found at the end of the intervention. However, we found a statistically significant improvement in quality of life, as measured by the Q-LES-Q-SF questionnaire, and a statistically significant decrease in the Anxiety item on the PANSS General Scale.

**Conclusions:**

Befriending was identified as providing an opportunity for increased social interactions and the development of healthy social relationships, suggesting that it may be considered a complementary or supplementary intervention for patients with schizophrenia, especially when CBTp is not readily available. A protocol involving the use of befriending as a pre-CBT intervention tool was suggested as a preparatory stage addressing social and interactional skills necessary for the more involved therapeutic engagement of the CBTp.

## Background

Schizophrenia is a severe, chronic mental disorder that involves disruptions in cognitive processes, emotional responsiveness, and social interactions. Individuals with schizophrenia tend to exhibit poor social functioning and are characteristically more socially isolated when compared to other groups of people in the general population (1). Some of the most commonly cited psychotherapeutic and social integration practices included in therapeutic guidelines, aside from pharmacological treatment, address these issues. These practices include CBT and cognitive-based interventions, social skills training, family interventions, and befriending, among others (2). Although not superior to any other intervention, befriending is viewed in the literature as one of the most readily available approaches within the healthcare system. Defined as a one-on-one companionship provided by a volunteer who aims to act as an emotionally supportive liaison (3), it requires no extensive training and poses no associated risks. Befriending has been described as distinct from mentoring, which aims at achieving specific goals. It is also viewed as distinct from peer support, where the person providing the befriending has lived experience. No particular dilemmas or symptoms are targeted during befriending sessions, and no deeper, private, mutual connection is implied. The topics discussed are generally of a neutral nature, including the client’s interests and hobbies. Delusions would not be challenged; instead, the conversation would be diverted to a neutral topic. Thus, the volunteer would not challenge any delusions of patients with schizophrenia and would instead divert the conversation to a different, neutral topic (2, 4). Due to the ease of initiating and low cost involved, befriending has the potential to be widely used in all clinical and community settings for patients with schizophrenia.

While befriending may have been exercised between patients and healthcare workers or volunteers for quite some time already, the effects of befriending on individuals with schizophrenia and their symptoms have not received extensive academic attention. In a systematic review of studies occurring before 2009 (5), only one literature source was identified that was at all related to befriending in treating early psychosis, and it was only used as a ***control condition*** to study the efficacy of CBT for psychosis.

After 2019, 4 studies that considered Befriending as an intervention in people living with schizophrenia consistently found improvements in some of the clinical or social domains. Although different protocols and different outcome measures were involved, their results confirm that befriending improves psychosocial and social functioning (6–8).

Improvements in certain clinical or social domains have been consistently observed in studies that incorporated befriending as an intervention (1, 9–11). Bozzattello et al. (9) found a significant improvement in psychosocial functioning, self-esteem and thought disturbance. In the Priebe et al. (1) study, participants in the befriending group had significantly more social contacts than those in the group offered information only. Similarly, Sikira et al. (10) found that the quality of life of participants in the befriending intervention significantly improved both at the endpoint and at the 12-month follow-up. Although different outcome measures were employed in these studies, their results align with the general view in the literature that befriending improves psychological and social functioning by reducing loneliness, allowing individuals to regain confidence and reconnect with social sources of support (12).

### Objectives

The primary objective of this study was to assess clinical changes in symptomatology using the Positive and Negative Syndrome Scale (PANSS), Cognitive Flexibility Scale (CFS) (13) and the Negative Symptom Assessment (NSA-16) (14, 15) in participants with schizophrenia undergoing a non-specific intervention, Befriending, along with their usual medication regimen.

Secondary Objectives. The secondary objectives were to assess changes in the quality of life and level of functioning before and after Befriending.

### Hypothesis

We hypothesized that befriending (cost-effective and easy-to-implement intervention) may have a positive impact on participant’s clinical presentation supporting readiness for therapy.

### Ethics statement

This research was reviewed by Queen’s University Health Sciences and Affiliated Teaching Hospitals Research Ethics Board (HSREB), as a part of the Research project 369826, 2019 PCC Rsh Innovation Grant, TRAQ DSS #6030572 titled “Innovative Pathways to Impactful Treatment Of Chronic Schizophrenia: Disrupting The Status-Quo Moving Toward Biologically-Driven, Combined Pharmacological And Non-Pharmacological Therapeutic Approaches To Define Markers Of Therapeutic Improvement In Cognitive Behavioral Therapy For Psychosis Promoted Recovery”.

## Methods

The purpose of this study was to assess the questionnaire-based correlates of recovery in individuals with schizophrenia and schizoaffective disorder who were undergoing a non-specific talk intervention called Befriending at Providence Care Hospital in Kingston, Ontario, Canada.

This is a prospective, repeated-measures study design. Patients with schizophrenia were treated with their usual medication regimen, unchanged for at least 3 months before enrolment in this study. After the screening visit, eligible subjects were exposed to a non-specific intervention, namely Befriending, for 4 weeks, with one Befriending session per week.

### Participants

Individuals with a diagnosis of Schizophrenia (DSM V criteria) stratified by age and sex, were recruited at Providence Care, Mental Health Services, in Kingston. Recruitment drew upon outpatient referral networks and community-based advertising. We expected 150 potentially eligible patients from PCH-MHS. Of these, 50 patients consented to participate, 40 were able to continue their involvement in this research, and only 32 completed the study. The inclusion and exclusion criteria are described in **Table 1**.

**Table 1:** Inclusion and exclusion criteria.

Inclusion criteria	Exclusion criteria
Diagnosis of SZ and SZA (DSM V criteria)	Moderate and severe comorbid intellectual disability
Stable under current treatment for at least 3 months (no recent changes in neuroleptics)	Current suicidal ideation/plans, treatment with stimulants, metronidazole, currently undergoing hormone therapy
Age over 18-65 (participants 65+ eligible depending on performance on cognitive assessment)	Age under 18
Able to provide written informed consent and, where not possible, by proxy	Unable to provide informed consent
Ability to understand English with reading level at or above grade 6	Not fluent in English
Able to understand and comply with the requirements of the study	Current illicit drug/substance abuse
Not taking benzos (hold) the morning of the intervention/investigations	History of TBI, MI, seizures, congenital heart disease, terminal/severe diseases (liver, kidney, cancer)
	Current enrollment in other formalized psychosocial intervention

### Experimental design, procedures and timelines

Our repeated-measures design consisted of the following visits: Visit 1 (screening, Week 0); Visit 2 (baseline, starting befriending, Week 1); and Visit 3 (end of befriending, Week 5), as shown in **Table 2**.

**Table 2:** Study visit schedule, procedures and timeline.

Timeline Procedure/Duration	Visit 1 Screening Week 0	Visit 2 Baseline Week 1	Visit 3 Session 1 Week 5
***Informed consent/60 min***	*X*		
*Chart review/1 hour*	*X*		
*MoCA/10 min*	*X*		
*C-SSRS/5 min*	*X*		
*BBTS/5 min*		*X*	
*PANNS/45 min*	*X*	*X*	*X*
*NSA/15 min*		*X*	*X*
*CFS/5 min*		*X*	*X*
*PSS/5 min*		*X*	*X*
*Q-LES-Q-SF/5 min*		*X*	*X*

The chart review searched for diagnosis, substance use, type of medication, side effects, and psychiatric family history.

### Psychosocial assessments

Several instruments were used to gain data concerning clinical symptoms, quality of life, level of functioning changes, personal history of trauma, and compliance with medication.

Primary outcome measures: the Positive and Negative Syndrome Scale (PANSS), the Negative Symptom Assessment (NSA 16 and NSA 4) and the Cognitive Flexibility Scale (CFS).

Secondary psychological measures: The Quality-of-Life Enjoyment and Satisfaction Questionnaire Short Form (Q-Les-Q-SF) (16), Brief Betrayal Trauma Survey (BBTS), and the Perceived Stress Scale (PSS) (17).

Screening instruments include the Montreal Cognitive Assessment (MoCA) and the Columbia Suicide Severity Rating Scale (C-SSRS) (18).

### Procedures

At the Screening Visit (visit 1), eligible participants provided written, informed consent for study procedures. Each patient underwent screening evaluations that included a full psychiatric consultation to confirm a diagnosis of Schizophrenia but also special evaluations with the C-SSRS scale (for all participants) and MoCA (for participants over the age of 65). The chart review searched for comorbid metabolic conditions, lab work abnormalities, substance use, BMI, type of medication, side effects, and family history.

### Intervention

The intervention consisted of befriending, comprising four individual-based, weekly sessions, each lasting 45–60 minutes.

The befriending sessions were provided by summer university students on placement with our hospital. The training was based on Carl Rogers’ three core conditions needed to create a safe and a nurturing space, where clients can be heard and understood. These students received one week of training on communication and social skills, active listening, safety, and boundaries, along with weekly debriefing support sessions throughout the intervention. Conceptualized as informal one-on-one interactions taking place at various out-of-home locations – parks, coffee shops, and libraries, these sessions went on for 45 minutes to an hour. Participants were encouraged to discuss topics meaningful to them, with the understanding that clinical aspects would be addressed during therapy. The discussed topics included finding common ground (exploring shared interests, hobbies and experiences that could help build a stronger connection), personal stories and memories (past events, family history, or milestones), current events and news (local happenings, or interesting news stories can spark conversation and create a sense of shared experience).

Expected outcomes: Participants will show improvement in psychosocial assessment scores at the end of the 4 weeks intervention.

The timeline for recruitment and intervention was 5–6 weeks.

## Results

### Participants

Over 150 participants were pre-screened, searching for eligibility criteria from the PCH-MHS existing database. From these, we selected 40 participants with schizophrenia (SZ) or schizoaffective disorder (SZA). Participants initially pre-screened and subsequently not enrolled in the study were excluded because they did not meet the inclusion and/or exclusion criteria (60%) or declined to participate (40%) for various reasons, such as being unable to attend all the visits for assessment and/or intervention.

The 40 participants with schizophrenia (SZ) or schizoaffective disorder (SZA) were screened during the first visit. Six of them did not meet the inclusion criteria for intervention. From the 34 initially chosen participants, one was discontinued due to the worsening symptoms after the Befriending session 3, and another client moved out of town and was unable to continue the intervention.

Thirty-two participants enrolled finished the study.

### Demographics/chart review

The demographics of the 32 participants are presented in **Table 3**.

**Table 3:** Demographics.

Variable		
	Number	%
Gender		
*Male*	22	68.75%
*Female*	10	31.25%
Race		
*White*	30	93.75%
*Indigenous*	1	3.13%
*East Asian*	1	3.13%
Education		
*< High school*	10	31.25%
*High school*	8	25%
*College*	7	21.88%
*University*	7	21.88%
Age		
*18-30*	1	3.13%
*31-55*	14	43.75%
*>55*	16	50%
Housing		
*Independent*	21	65.63%
*With a roommate*	1	3.13%
*With family*	5	15.63%
*Supported housing*	5	15.63%
Employment		
*Supported employment*	7	21.88%
*Volunteer*	2	6.25%
*Part-time employment*	4	12.5%
*No employment*	19	59.38%
Employment history		
*No*	7	21.8 8%
*Yes*	25	78.13%
Financial		
*ODSP*	20	62.5%
*ODSP/CPP*	3	9.38%
*ODSP/Employment income*	6	18.75%
*CPP/Old age pension*	3	9.38%
Family support		
*No*	10	31.25%
*Yes*	22	68.75%
Partner		
*No*	26	81.25%
*Yes*	6	18.75%
Friends		
*No*	13	40.63%
*Few*	14	43.75%
*Many*	5	15.63%

The psychiatric diagnoses, psychiatric history (age of onset, number of hospitalizations), personal history of trauma and use of substances, as well as family history, are represented in **Table 4**.

**Table 4:** Psychiatric and medical conditions.

Variable		
	Number	%
Main psychiatric diagnosis		
*Schizophrenia*	18	56.25%
*Schizoaffective Disorder*	14	43.75%
Age at onset		
*< 25 years old*	18	56.25%
*26–40 years old*	14	43.75%
Number of hospitalizations		
*< 5 times*	18	56.25%
*6–10 times*	11	34.38%
> 10 times		
*Trauma*	13	40.63%
Drug use history		
*Alcohol*	6	18.75%
*Alcohol, cannabis, illegal drugs*	10	31.25%
*Alcohol, cannabis, illegal drugs, medication*	2	6.25%
*None*	12	37.5%
Current drug use		
*Cannabis, Nicotine*	3	9.38%
*Nicotine*	13	40.63%
Psychiatric family history		
*Schizophrenia/Schizoaffective Disorder*	16	50.0%
*Affective Disorders*	3	9.38%
*Substance use*	11	34.38%
*Suicide*	8	25%

The Befriending was an add-on intervention to the usual medication regimen, presented in **Table 5**.

**Table 5:** Current medication regimen.

Variable		
Current Antipsychotic Medication		
*Clozapine*	9	28.13%
*Quetiapine*	4	12.5%
*Olanzapine*	5	15.63%
Aripiprazole		
*Paliperidone*	3	9.38%
*Asenapine*	1	3.13%
*Brexpiprazole*	1	3.13%
*Risperidone*	1	3.13%
Combination of antipsychotics		
*Olanzapine + Aripiprazole*	2	6.25%
*Olanzapine + Chlorpromazine*	1	3.13%
*Quetiapine + Aripiprazole*	1	3.13%
*Quetiapine + Lurasidone + Chlorpromazine*	1	3.13%
*Aripiprazole + Paliperidone*	1	3.13%
*Long-acting antipsychotics*	9	28.13%
*Antidepressive medication*	8	25%
*Mood stabilizer*	2	6.25%

### Befriending

The Befriending was conducted by undergrad students, specifically and intensively trained for 14 days to apply the protocol developed for the study. This intervention was provided to all eligible participants (N = 32).

A Wilcoxon signed rank test was performed to reveal changes between baseline and post-befriending scores, with statistically significant results for the Q-LES-Q-SF and the Anxiety item of the PANSS General. The Wilcoxon signed rank test examines information on the differences and on the magnitude of difference between 2 studied parameters (before and after procedure), being the most powerful “sign test”.

The means and standard deviations for the befriending (pre- and post-) primary outcomes are displayed in **Table 6**. The results are presented for the entire sample, providing intent-to-treat scores as post-treatment outcomes.

**Table 6:** Befriending outcome measures.

Outcome measures	Baseline		Post-Befriending		Wilcoxon	
	Median	Mean	Median	Mean	Z	P
Q-LES-Q-SF	37.00	37.78	41.50	41.65	4.37	.001
Mood	2.50	2.50	3.00	2.87	2.81	.005
Social Relationships	2.00	1.97	2.00	2.47	3.55	.001
Family Relations	2.00	1.87	2.00	2.63	3.51	.001
Ability to function in daily life	3.00	2.59	3.00	3.03	2.88	.004
Leisure time activities	2.00	1.88	3.00	2.66	3.80	.001
Overall Sense of Wellbeing	3.00	2.53	3.00	2.94	2.70	.007
CFS	35.00	35.00	35.00	35.00	.059	.953
PANSS Total	92.00	93.56	89.50	90.84	1.48	.138
PANSS General	47.00	48.34	45.00	46.59	1.65	.099
PANSS General Anxiety	4.00	3.78	4.00	3.59	2.12	.034
NSA-16 Global Score	4.00	3.88	4.00	3.75	1.63	.102
PSS	25.00	25.59	23.00	24.31	1.58	.113

## Discussion

We found statistically significant improvements in quality of life among participants who received four weekly sessions of befriending. The most significant dimensions showing improvement, as assessed with the Q-LES-Q-SF, were mood, social relationships, family relationships, ability to function in daily life, leisure activities, and an overall sense of well-being. In one of the earliest studies on the therapeutic impact of befriending in schizophrenia, Sikira et al. (10) found that the quality of life of participants in the befriending intervention significantly improved both at the endpoint and at the 12-month follow-up. In the 5-year follow-up to this study, Turkington et al. (19, 20) found that although both groups (CBT and Befriending) showed a clinical improvement, only the CBT group showed a significant difference over time, concluding that CBT is superior to befriending in reducing psychotic symptoms.

We found no clinical improvement resulting from the befriending intervention in our study, as assessed by the clinical scales, except for the PANSS General Anxiety dimension. We found a statistically significant decrease in the PANSS General Anxiety dimension from pre- to post-befriending, *as indicated by a Z score of 2.12 and a p-value of 0.034* (**Table 5**). A statistically significant reduction in anxiety appears to sustain the statistically significant improvement in quality of life observed (*Z* = 4.37, *p* = 0.001, as seen in **Table 5**).

Although extensive literature highlights the effectiveness and superiority of CBT for psychosis by comparison with the befriending intervention, it is worth acknowledging the benefits an intervention like befriending can provide, considering its cost advantage, acceptability and level of satisfaction reported. The decreased anxiety level and learning to secure a structured time for a structured interaction once per week make befriending an efficient pre-CBT for psychosis tool, preparing the clients for it. While highly recommended by clinical practice guidelines, psychotherapeutic interventions such as CBT for psychosis require lengthy therapist training and prove challenging to be delivered on a large scale within mental health services. Befriending could be used as a sole intervention in cases where CBT for psychosis is not an option or is not recommended, providing social engagement and acceptance for this stigmatized population segment in the context of a comprehensive socio-psychopharmacological approach.

## Conclusions

In our study, we did not find a statistically significant clinical improvement at the end of the intervention in people living with schizophrenia, undergoing 4 weeks of weekly Befriending sessions. However, we found a statistically significant improvement in their quality of life, as measured by the Q-LES-Q-SF questionnaire, and a statistically significant decrease in the Anxiety item on the PANSS General Scale. Befriending was identified as providing an opportunity for increased social interactions and the development of healthy social relationships, suggesting that it may be considered a complementary or supplementary intervention for patients with schizophrenia, especially when CBT for psychosis is not readily available. A protocol involving the use of befriending as a pre-CBT intervention tool was suggested as a preparatory stage addressing social and interactional skills necessary for the more involved therapeutic engagement of the CBTp.

### Limitations

include a small sample size, the absence of a control group, and no follow-ups after befriending. We didn’t have follow-up because the group of participants completed further a subsequent study, involving CBT for psychosis, Befriending being the first step of a new psychological interventional protocol, with the use of befriending as a *pre-intervention tool*, preparing patients for CBT for psychosis.

### Further directions

Further directions pertain to *larger sample size studies* targeting befriending in schizophrenia, with well-defined protocols and longer follow-ups to determine the timing and frequency of maintenance sessions. These studies should utilize the most relevant clinical outcome measures, including the PANSS, QoL, and scales assessing third-party or collateral information. Based on the results from follow-ups, we could design possible *intervention protocols* that include well-timed maintenance sessions. A possible model of a maintenance session could be based on the protocol used in the Bottero-Rodriguez et al. (9) study and include a progression from one-on-one to a small group format. These protocols would be easy to implement in all clinical and community settings, as well as cost-effective, provided that the intervention is not performed by highly trained clinicians. The next step in this regard may involve developing a minimal yet necessary *training curriculum* for volunteers. This would ensure that volunteers do not interfere with or challenge problematic symptoms while at the same time allowing them to interact with patients in a non-judgmental, accepting and hope-promoting manner.

An avenue not explored in the literature is the use of befriending as a *pre-intervention tool*, preparing patients for accepted interventions, such as CBT. The interactions with a volunteer in the time leading to the clinical intervention would open the door to the therapeutic engagement essential for therapeutic change. In this context, adequate resources would be allocated to achieve appropriate outcomes and ensure a more comprehensive and effective approach.

## Data Availability

The raw data supporting the conclusions of this article will be made available by the authors, without undue reservation.
